# Supplementary material for: Antiviral responses in a Jamaican fruit bat intestinal organoid model of SARS-CoV-2 infection
Source: Nat Commun. 2023 Oct 28;14:6882. doi: 10.1038/s41467-023-42610-x (PMC10613288; doi:10.1038/s41467-023-42610-x)
Supplement: Supplementary file 1 — Supplementary Information [file 41467_2023_42610_MOESM1_ESM.pdf]

Supplementary Figures and Tables

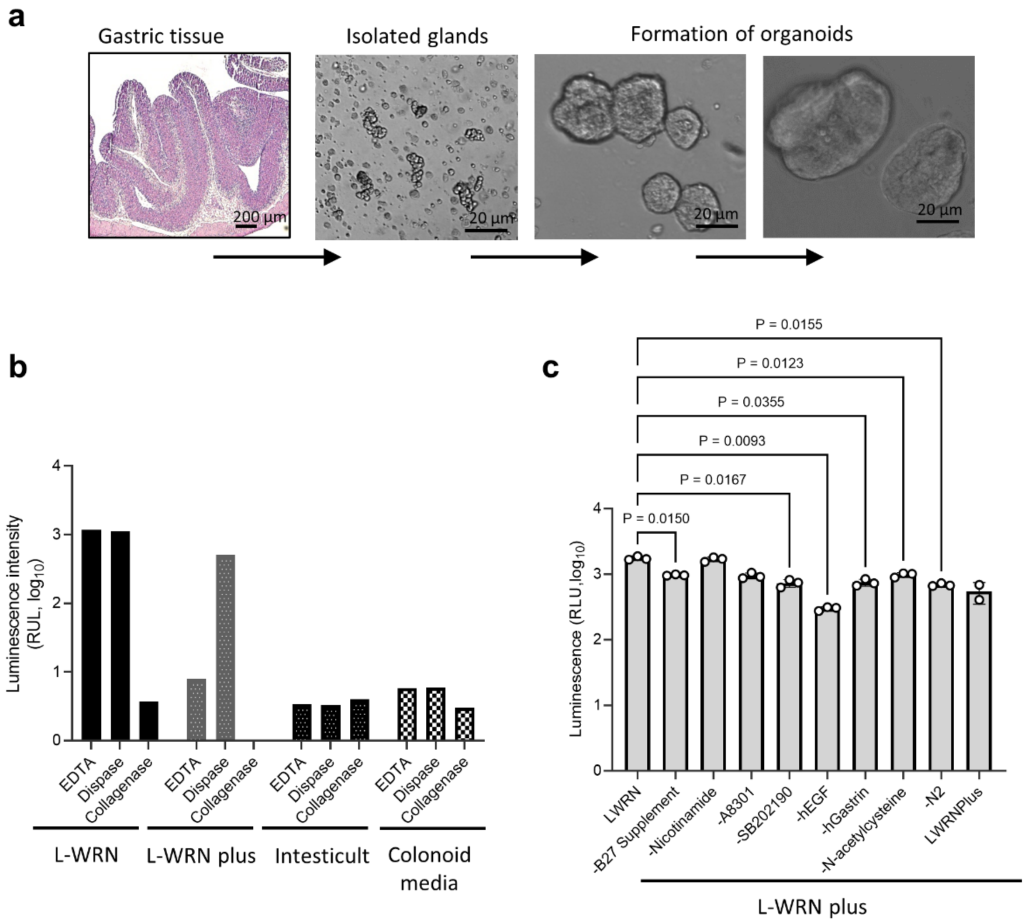

Supplementary Figure 1: **Development and characterization of gastrointestinal organoids from Jamaican fruit bats**. (**a**) Gastric tissue from a Jamaican fruit bat was incubated for 60 min in collagenase IV solution to isolate glands. The isolated glands were resuspended in Matrigel, seeded in a 96-well plate and cultured with L-WRN media. Organoid formation was observed 24 hours later. (**b**) JFB distal intestinal tissue was digested in EDTA solution, Dispase® or collagenase to isolate crypts. For Dispase® digestion, the minced tissue was placed in 1-2 mL of Dispase® (Gibco) and incubated on ice for up to 30 min with periodic gentle shaking until crypts appeared in supernatant. The isolated crypts were resuspended in Matrigel, seeded in a 96-well plate and then were overlaid with L-WRN media, L-WRN plus media, IntestiCult™ or colonoid media. After four days, organoid proliferation was analyzed using the CellTiter-Glo Luminescence assay. One representative out of three experiments with n=1 wells per condition. (**c**) Growth conditions were further optimized by eliminating one additive/growth factor from the L-WRN plus media at a time, n=3 technical replicates, mean ± SD, representative of three independent experiments. Data were analyzed using One-way ANOVA with Tukey's multiple comparisons.

Noggin

```
Mus musculus           MERCPSLGVTLYALVVVLGLRAAPAGGQHYLHIRPAPSDNLPLVDLIEHPDPIFDPKEKDLNETLLRSLLGGHYDPGFMA
Artibeus jamaicensis   MDRCPSLGVTLYALVVVLGLRAAPAGGQHYLHIRPAPSDNLPLVDLIEHPDPIFDPKEKDLNETLLRSLLGGHYDPGFMA
                       *:******************************************************************************

Mus musculus           TSPPEDRPGGGGGPAGGAEDLAELDQLLRQRPSGAMPSEIKGLEFSEGLAQGKKQRLSKKLRRKLQMWLWSQTFCPVLYA
Artibeus jamaicensis   TSPPEDRPGGGGGSAGSAEDLAELDQLLRQRPSGAMPSEIKGLEFSEGLAPGKKQRLSKKLRRKLQMWLWSQTFCPVLYA
                       *************.**.********************************* *****************************

Mus musculus           WNDLGSRFWPRYVKVGSCFSKRSCSVPEGMVCKPSKSVHLTVLRWCQRRGGQRCGWIPIQYPIISECKCSC
Artibeus jamaicensis   WNDLGSRFWPRYVKVGSCFSKRSCSVPEGMVCKPSKSVHLTVLRWCQRRGGQRCGWIPIQYPIISECKCSC
                       ***********************************************************************
```

R-spondin

```
Mus musculus           MRLGLCVVALVLSWTHIAVGSRGIKGKRQRRISAEGSQACAKGCELCSEVNGCLKCSPKLFILLERNDIRQVGVCLPSCP
Artibeus jamaicensis   MRLGLCVVALVLSWMHLTAGSRGTKGKRQRRISAEGSQACAKGCELCSEVNGCLKCSPKLFILLERNDIRQVGVCLPSCP
                       ************** *::.**** ********************************************************

Mus musculus           PGYFDARNPDMNKCIKCKIEHCEACFSHNFCTKCQEGLYLHKGRCYPACPEGSTAANSTMECGSPAQCEMSEWSPWGPCS
Artibeus jamaicensis   PGYFDARNPDMNKCIKCKIEHCEACFSHNFCTKCQESLYLHKGRCYLTCPEGSTAANGTMECSSPPQCEMTEWSPWGPCT
                       ************************************.********* :*********.****.**.****:********:

Mus musculus           KKRKLCGFRKGSEERTRRVLHAPGGDHTTCSDTKETRKCTVRRTPCPEGQKRRKGGQGRRENANRHPARKNSKEPGSNSR
Artibeus jamaicensis   KKKKTCGFRRGSEERTRRVLQAPGGDHAVCSDTKEIRRCTVRRTPCPEGQKRRKGGQGRRENANRNASRKESKEAGTGSR
                       **:* ****:**********:******:.****** *:***************************:.:**:***.*:.**

Mus musculus           RHKGQQ---QPQPGTTGPLTSVGPTWAQ
Artibeus jamaicensis   RRKGKQQQQQQQQGTVGPLTSAGPT---
                       *:**:*   * * **.*****.***
```

Wnt3a

```
Mus musculus           MEPHLLGLLLGLLLCGTGVLAGYPIWWSLALGQQYTSLGSQPLLCGSIPGLVPKQLRFCRNYIEIMPSVAEGVKLGIQEC
Artibeus jamaicensis   MEPHLLGLLLGLLLSGTRVLAGYPIWWSLALGQQYTSLASQPLLCGSIPGLVPKQLRFCRNYIEIMPSVAEGVKLGIQEC
                       **************.** ********************.*****************************************

Mus musculus           QHQFRGRRWNCTTIDDSLAIFGPVLDKATRESAFVHAIASAGVAFAVTRSCAEGTSTICGCDSHHKGPPGEGWKWGGCSE
Artibeus jamaicensis   QHQFRGRRWNCTTIDDSLAIFGPVLDKATRESAFVHAIASAGVAFAVTRSCAEGTSTICGCDSHHKGPPGEGWKWGGCSE
                       ********************************************************************************

Mus musculus           DADFGVLVSREFADARENRPDARSAMNKHNNEAGRTTILDHMHLKCKCHGLSGSCEVKTCWWAQPDFRAIGDFLKDKYDS
Artibeus jamaicensis   DADFGVLVSREFADARENRPDARSAMNKHNNEAGRTTILDHMHLKCKCHGLSGSCEVKTCWWAQPDFRAIGDFLKDKYDS
                       ********************************************************************************

Mus musculus           ASEMVVEKHRESRGWVETLRAKYALFKPPTERDLVYYENSPNFCEPNPETGSFGTRDRTCNVTSHGIDGCDLLCCGRGHN
Artibeus jamaicensis   ASEMVVEKHRESRGWVETLRAKYALFKPPTERDLVYYENSPNFCEPNPETGSFGTRDRTCNVTSHGIDGCDLLCCGRGHN
                       ********************************************************************************

Mus musculus           TRTEKRKEKCHCIFHWCCYVSCQECVRIYDVHTCK
Artibeus jamaicensis   TRTEKRKEKCHCVFHWCCYVSCQECIRIYDVHTCK
                       ************:************:********
```

__Supplementary Figure 2:__ **Protein sequence alignment of murine and *Artibeus jamaicensis* growth factors.** Protein sequence alignment of murine and *Artibeus jamaicensis* (taxid:9417) Noggin, R-spondin, and Wnt3a was performed using T-Coffee, a multiple sequence alignment web server [1]. Noggin had 98% positive identity with 98% coverage, R-spondin has 86% positive identity and 98% coverage, and Wnt3 had 99% positive identity with 90% coverage. [*] Same AA; [:] and yellow highlight: conservative AA replacement; [.] and red highlight: semi-conservative AA replacement; space and cyan highlight: non-conservative AA replacement. The gap in the R-spondin sequence is highlighted in dark red.

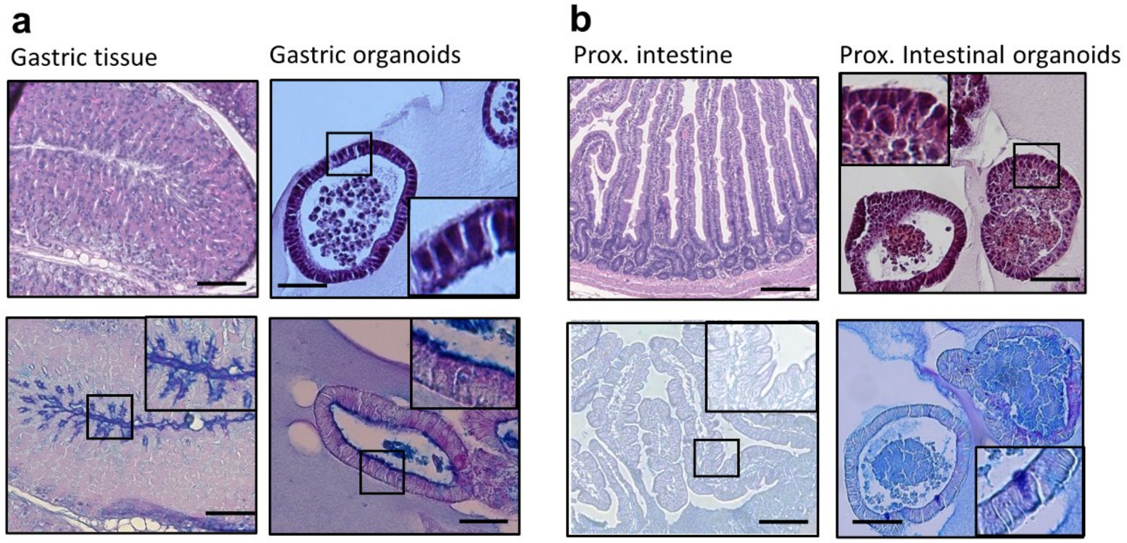

<u>Supplementary Figure 3:</u> Histological analysis of tissue and organoids from JFB stomach and proximal intestine. Morphology of (**a**) JFB stomach tissue and organoids and (**b**) proximal intestinal tissue and organoids. Formalin-fixed, paraffin-embedded sections were stained with hematoxylin and eosin (top row) or Alcian Blue to identify mucus (bottom row). High magnification insets show columnar cell shape and morphology of mucus-secreting goblet cells. Representative images out of four tissue samples and organoid lines are shown.

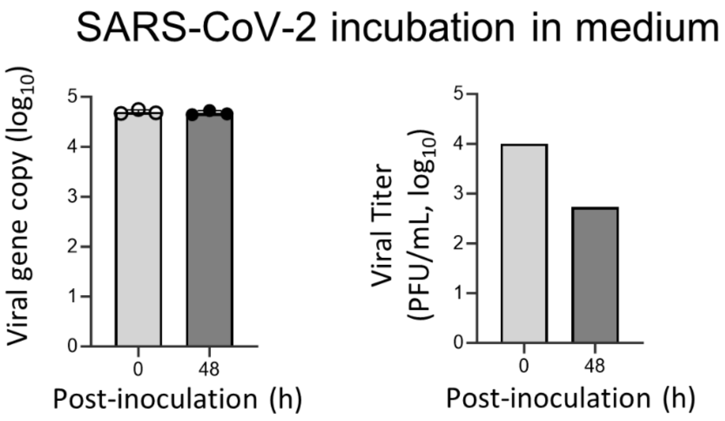

Supplementary Figure 4: **SARS-CoV-2 loses infectivity in culture medium**. SARS-CoV-2 was resuspended in medium and at time 0 h and 48 h post infection, the medium was collected, and viral RNA was extracted using QIAmp Viral RNA mini kit (Qiagen). SARS-CoV-2 RNA was measured via quantitative real-time PCR for the E gene (left). Plaque assay was also performed on these supernatants (right). The experiment was performed once, with three technical replicates for the PCR and one well for plaque assays.

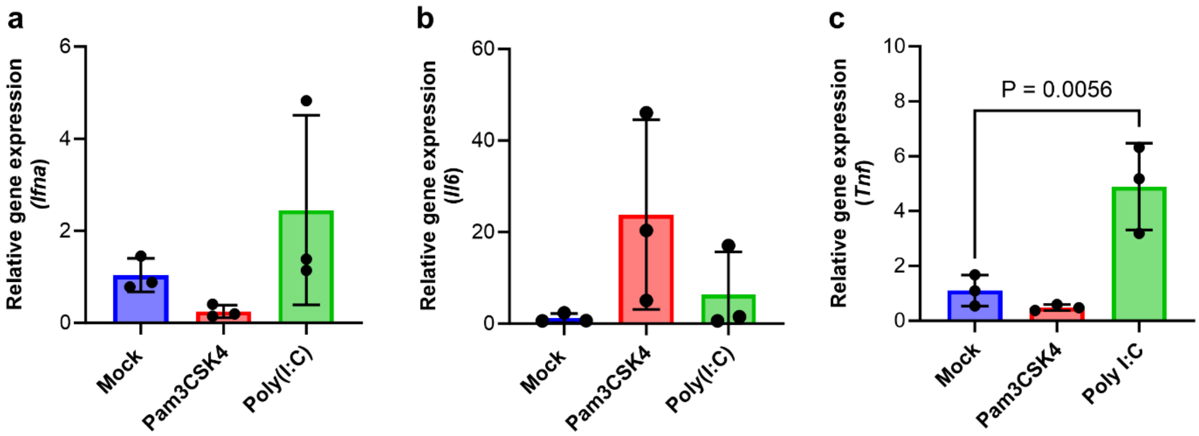

<u>Supplementary Figure 5</u>: Gene expression of JFB organoids following short term TLR stimulation. Organoids were treated with the TLR2/1 agonist Pam3CSK4 or the TLR3 agonist low MW poly I:C for 6 h and then were analyzed by qRT-PCR for expression of (**a**) *Ifna*, (**b**) *Il6*, (**c**) *Tnf*. Three technical replicates with mean ± SD, representative of n=6 (*Ifna)* or n=4 (*Tnf*, *Il6*) independent experiments are shown. Data were analyzed by one-way ANOVA with Dunnett's multiple comparison test.

Supplementary Table 1: Composition of culture media for JFB gastrointestinal organoids

	Concentration	Supplier
**<u>L-WRN Media</u>**		
Advanced DMEM/F12		Gibco by Life Technology #12-491-015
HEPES	10 mM	Cytiva Hyclone #SH3023701
Pen/Strep	1 %	Cytiva Hyclone #SV30010
L-WRN conditioned supernatant	50 %	In house, cells kindly provided by Dr. T. Stappenbeck. ATCC #CRL-3276
ROCK-inhibitor (Y-2732)	10 M	Tocris Bioscience #125410
Amphotericin B	10 µM	Fisher Scientific #BP928-250
Gentamycin	1 %	IBI Scientific #IB02030
L-Glutamine	1 %	Cytive # SH30034.01
TGF-β inhibitor (SB-431542)	10 µM	Tocris Bioscience #161410
FBS	10 %	Atlas Biologicals #F-0500-D
**<u>L-WRN-Plus media</u>**		
L-WRN media (as above)		
B27 Supplement	1x	Gibco by Life Technology #A-3582801
Nicotinamide	10 mM	Thermo Scientific #AC128275000
A8301	500 nM	Tocris #2939/10
human epidermal growth factor (hEGF)	100 ng/mL	Abcam #AB28545850011G
N-acetylcysteine	1 mM	Sigma Aldrich #A9165-5G
**<u>IntestiCult™ Media</u>**		StemCell Technologies, #06005
**<u>Colonoid media</u>**		
Advanced DMEM/F12		Gibco by Life Technology #12-491-015
L-WRN-conditioned supernatant	50 %	In house, cells kindly provided by Dr. T. Stappenbeck. ATCC #CRL-3276
HEPES	10mM	Cytiva Hyclone #SH3023701
N2 Supplement	1x	Gibco by Life Technology #17502048
B27 Supplement	1x	Gibco by Life Technology #A3582801
Pen/Strep	1 %	Cytiva Hyclone #SV30010
N-acetylcysteine	2 mM	Sigma Aldrich #A9165-5G
Nicotnamide	10 mM	Thermo Scientific #AC128275000
human epidermal growth factor (hEGF)	100 ng/mL	Abcam #AB28545850011G

Supplementary Table 2: Primer and probe sequences

Gene Name	Sequence (5'-3')
**Stomach**	
Pgc	
**Forward**	CCCCTGAGAGAGTCAAGTGC
**Reverse**	TGGCACTTCTGAACAGGGTC
Muc5ac	
**Forward**	AGTGCAGGGCAAATCGTACA
**Reverse**	AGGCGTCTGCATCGTATGTG
**Small Intestine**	
Cdx2	
**Forward**	GCCAAGTGAAAACCAGGACG
**Reverse**	TCTCGGAGAGCCCCAGG
Vil1	
**Forward**	CTTGCCTGTGTGGAAGCAAC
**Reverse**	GGCTGCTCACAGGTACAACT
Muc2	
**Forward**	GTGGTCGTCTCCTACAACGG
**Reverse**	ATCCTGCGTGTTGTTGTTGC
Ace2	
**Forward**	ACTAAGCCAGATGATGGCCG
**Reverse**	GTAGGAAGGGTTGGTTGGCA
**Immune Genes**	
Ifna4l* and *IFNa10l	
**Forward**	ACAAATGAGAAGGACCGCCT
**Reverse**	GAACTGGTTGCCACTGAACG
Ifnb	
**Forward**	GAGCTACGATGTGCTTCGGT
**Reverse**	CATCCTGTCCTGGAGGCAAT
Tnf	
**Forward**	AATTGGCCCTTCACTGGCTC
**Reverse**	CCCACAGCTTGCTGATTTGC
Il6	
**Forward**	GACAATGCCAAGGCTATGCAG
**Reverse**	GTCTTCCTCCACTCGTTCTGG
**SARS-CoV-2**	
E gene / genomic	

**Forward**	ACAGGTACGTTAATAGTTAATAGCGT
**Reverse**	ATATTGCAGCAGTACGCACACA
**Probe**	FAM-ACACTAGCCATCCTTACTGCGCTTCG-BHQ1

**Gene Name**	**Sequence (5’-3’)**
E gene / subgenomic	
**Forward**	CGATCTCTTGTAGATCTGTTCTC
**Reverse**	ATATTGCAGCAGTACGCACACA
**Probe**	FAM-ACACTAGCCATCCTTACTGCGCTTCG-BHQ1

Supplementary Data 1:

Excel spreadsheet. All identified proteins with significantly increased or decreased expression upon infection with SARS-CoV-2 for 48 h at an MOI=10 ($P \leq 0.05$).

Supplementary Data 2:

Excel spreadsheet. List of all interferon-stimulated genes (ISGs) identified in Jamaican fruit bat distal small intestinal organoids based on a comparison with a human ISG list compiled by OhAinle et al. [2].

Supplementary References

1. Notredame C, Higgins DG, Heringa J. T-Coffee: A novel method for fast and accurate multiple sequence alignment. *J Mol Biol* **302**, 205-217 (2000), doi: 10.1006/jmbi.2000.4042.

2. OhAinle M*, et al.* A virus-packageable CRISPR screen identifies host factors mediating interferon inhibition of HIV. *Elife* **7**, (2018), doi: 10.7554/eLife.39823.
