# Supplementary material for: Antiviral responses in a Jamaican fruit bat intestinal organoid model of SARS-CoV-2 infection
Source: Nat Commun. 2023 Oct 28;14:6882. doi: 10.1038/s41467-023-42610-x (PMC10613288; doi:10.1038/s41467-023-42610-x)
Supplement: Supplementary file 3 — Description of Additional Supplementary Files [file 41467_2023_42610_MOESM3_ESM.pdf]

Description of Additional Supplementary Files

File Name: Supplementary Data 1
Description: Excel spreadsheet. All identified proteins with significantly increased or decreased expression upon infection with SARS-CoV-2 for 48 h at an MOI=10 ($P \leq 0.05$).

File Name: Supplementary Data 2
Description: Excel spreadsheet. List of all interferon-stimulated genes (ISGs) identified in Jamaican fruit bat distal small intestinal organoids based on a comparison with a human ISG list compiled by OhAinle et al.
