# Supplementary material for: Antiviral responses in a Jamaican fruit bat intestinal organoid model of SARS-CoV-2 infection
Source: Nat Commun. 2023 Oct 28;14:6882. doi: 10.1038/s41467-023-42610-x (PMC10613288; doi:10.1038/s41467-023-42610-x)
Supplement: Supplementary file 6 — Reporting Summary [file 41467_2023_42610_MOESM6_ESM.pdf]

nature portfolio

Corresponding author(s): Diane Bimczok

Last updated by author(s): Sep 28, 2023

Reporting Summary

Nature Portfolio wishes to improve the reproducibility of the work that we publish. This form provides structure for consistency and transparency in reporting. For further information on Nature Portfolio policies, see our Editorial Policies and the Editorial Policy Checklist.

Statistics

For all statistical analyses, confirm that the following items are present in the figure legend, table legend, main text, or Methods section.

n/a	Confirmed	
☐	☒	The exact sample size (*n*) for each experimental group/condition, given as a discrete number and unit of measurement
☐	☒	A statement on whether measurements were taken from distinct samples or whether the same sample was measured repeatedly
☐	☒	The statistical test(s) used AND whether they are one- or two-sided<br>*Only common tests should be described solely by name; describe more complex techniques in the Methods section.*
☒	☐	A description of all covariates tested
☒	☐	A description of any assumptions or corrections, such as tests of normality and adjustment for multiple comparisons
☐	☒	A full description of the statistical parameters including central tendency (e.g. means) or other basic estimates (e.g. regression coefficient) AND variation (e.g. standard deviation) or associated estimates of uncertainty (e.g. confidence intervals)
☐	☒	For null hypothesis testing, the test statistic (e.g. F, t, r) with confidence intervals, effect sizes, degrees of freedom and P value noted<br>*Give P values as exact values whenever suitable.*
☒	☐	For Bayesian analysis, information on the choice of priors and Markov chain Monte Carlo settings
☒	☐	For hierarchical and complex designs, identification of the appropriate level for tests and full reporting of outcomes
☒	☐	Estimates of effect sizes (e.g. Cohen's *d*, Pearson's *r*), indicating how they were calculated

Our web collection on statistics for biologists contains articles on many of the points above.

Software and code

Policy information about availability of computer code

Data collection	PCR data was collected using QuantStudio(TM) Design&Analysis Software v1.5.1.<br>Confocal Images were collected using Leica LAS X imaging software, v 4.5.0. or earlier.<br>Fluorescent, brightfield and phase contrast imaged were collected using the Keyence BZ-X800 Viewer software, v01.02.03.02, or with a Life Technologies EVOS FL Auto System.<br>Electron micrographs were collected using a LEO 912AB TEM with integrated software.
Data analysis	Data analysis was performed using Microsoft Office Excel and GraphPad Prism 9.5.1. or earlier versions. Image data were analyzed using ImageJ 1.5.3t or earlier versions. Organoid morphometry was performed using OrganoSeg, a MATLAB-based stand alone software for organoid analysis described by Borten et al., 2018 (DOI: 10.1038/s41598-017-18815-8).<br>Proteome data was analyzed using the following tools: Prosit, EncyclopeDIA, ProteiNorm, Scaffold DIA 3.3.1, Ingenuity Pathway Analysis, and Enrichr.

For manuscripts utilizing custom algorithms or software that are central to the research but not yet described in published literature, software must be made available to editors and reviewers. We strongly encourage code deposition in a community repository (e.g. GitHub). See the Nature Portfolio guidelines for submitting code & software for further information.

Data

Policy information about availability of data

All manuscripts must include a data availability statement. This statement should provide the following information, where applicable:

- Accession codes, unique identifiers, or web links for publicly available datasets
- A description of any restrictions on data availability
- For clinical datasets or third party data, please ensure that the statement adheres to our policy

The mass spectrometry proteomics data have been deposited to the ProteomeXchange Consortium via the PRIDE partner repository with the dataset identifier PXD036016. The raw data underlying bar charts and scatter plots that support the findings of this study are available in Figshare (https://doi.org/10.6084/m9.figshare.23536797). Raw imaging data are available from the corresponding author upon reasonable request. Relevant public databases queried by Enrichr are Human KEGG pathways 2021 (https://www.kegg.jp/) and COVID-19 Related Gene Sets 2021 (https://maayanlab.cloud/covid19/).

Research involving human participants, their data, or biological material

Policy information about studies with human participants or human data. See also policy information about sex, gender (identity/presentation), and sexual orientation and race, ethnicity and racism.

Reporting on sex and gender	N/A
Reporting on race, ethnicity, or other socially relevant groupings	N/A
Population characteristics	N/A
Recruitment	N/A
Ethics oversight	N/A

Note that full information on the approval of the study protocol must also be provided in the manuscript.

Field-specific reporting

Please select the one below that is the best fit for your research. If you are not sure, read the appropriate sections before making your selection.

☒ Life sciences ☐ Behavioural & social sciences ☐ Ecological, evolutionary & environmental sciences

For a reference copy of the document with all sections, see nature.com/documents/nr-reporting-summary-flat.pdf

Life sciences study design

All studies must disclose on these points even when the disclosure is negative.

Sample size	No sample size calculation was performed. Experiments were performed with three to five organoid lines, as is customary in the field (see e.g. Guerts et al., Nature Communications 2023, Fig. 3; Danho et al. Gut Microbes 2023, Fig. 3; Hu, Xioa, Lei et al., Nature Communications 2023, Fig. 3).
Data exclusions	No data were excluded from the analyses.
Replication	At least three biological replicates or three independent experiments were analyzed. When experiments yielded variable results, as is common with primary cells from outbred species, data that were similar to the mean of all experiments were chosen as representative experiments.
Randomization	Randomization was achieved by pooling organoids from multiple wells together and then equally distributing them into multiple tubes or wells immediately before infection or treatment.
Blinding	For microscopic image analysis and plaque assays, the investigator was blinded to the treatment of the samples. Blinding was not relevant for other types of analyses, because all other experimental methods (PCR, proteomics, proliferation assays, transepithelial resistance) do not have any observer bias.

Reporting for specific materials, systems and methods

We require information from authors about some types of materials, experimental systems and methods used in many studies. Here, indicate whether each material, system or method listed is relevant to your study. If you are not sure if a list item applies to your research, read the appropriate section before selecting a response.

Materials & experimental systems

n/a	Involved in the study	
☐	☒	Antibodies
☐	☒	Eukaryotic cell lines
☒	☐	Palaeontology and archaeology
☐	☒	Animals and other organisms
☒	☐	Clinical data
☒	☐	Dual use research of concern
☒	☐	Plants

Methods

n/a	Involved in the study	
☒	☐	ChIP-seq
☒	☐	Flow cytometry
☒	☐	MRI-based neuroimaging

Antibodies

Antibodies used	(1) Anti-pan-cytokeratin, ThermoFisher Scientific, cat. #4545, clone C11, lot #14, used at 1:50<br>(2) Anti-villin, Invitrogen, cat. # MA5-16408, clone SP145, lot #XA3488001, used at 1 : 100<br>(3) Anti-ACE2, R&D Systems, cat. # AF933, goat polyclonal , lot #HOK0320051, used at 1 : 100<br>(4) Anti-SARS-CoV-2 (USA/WA1/2020), in house, clone 11G10-F8, used at 10 ug/mL
Validation	Validated antibodies for Jamaican fruit bat epithelial cell markers are not available from any manufacturer. Therefore, primary antibodies with reported reactivity across multiple different species were selected. Specificity of antibody binding to their target proteins was was confirmed based on the expected cellular distribution of target proteins. The anti-SARS-CoV-2 antibody was validated by ELISA assay using SARS-CoV-2 subunits (RBD, S1 and S2, from BEI) and found to bind to the RBD subunit.

Eukaryotic cell lines

Policy information about cell lines and Sex and Gender in Research

Cell line source(s)	Organoid lines generated from the stomach, proximal and distal intestine of five Jamaican Fruit Bats (Artibeus jamaicensis)<br>Lines from bat001, 002, 004 and 005 were derived from male bats, lines from bat003 was derived from a female bat.<br>VeroE6 cells were obtained from ATCC, #CRL-1586<br>L-WRN wells were kindly provided by Dr. T. Stappenbeck.
Authentication	Organoid lines were authenticated as part of this study using the methods described in the manuscript. L-WRN cells were authenticated by demonstrating Wnt3a production using a bioassay. VeroE6 cells were not authenticated.
Mycoplasma contamination	Randomized testing that included three out of five bat organoid lines found no mycoplasma. L-WRN cells and VeroE6 cells also tested negative for mycoplasma.
Commonly misidentified lines<br>(See ICLAC register)	No commonly misidentified cell lines were used.

Animals and other research organisms

Policy information about studies involving animals; ARRIVE guidelines recommended for reporting animal research, and Sex and Gender in Research

Laboratory animals	Male and female Jamaican fruit bats (Artibeus jamaicensis, 1 - 8 years) were used for this study.
Wild animals	No wild animals were used in this study.
Reporting on sex	Sex was not considered as part of the study design. Due to the small number of organoid lines (five) in this study, with only one derived from a female, statistical analyses based on sex were not feasible.
Field-collected samples	No field collected samples were used in this study.
Ethics oversight	Colorado State University IACUC, protocol 1034.

Note that full information on the approval of the study protocol must also be provided in the manuscript.
